# Maternal and health care workers’ perceptions of the effects of exclusive breastfeeding by HIV positive mothers on maternal and infant health in Blantyre, Malawi

**DOI:** 10.1186/1471-2393-14-247

**Published:** 2014-07-25

**Authors:** Ursula K Kafulafula, Mary K Hutchinson, Susan Gennaro, Sally Guttmacher

**Affiliations:** Kamuzu College of Nursing, P.O. Box 415, Blantyre, Malawi; New York University School of Nursing, 726 Broadway, 10th Floor, New York, NY 10003 USA; Boston College, Connell School of Nursing, Chestnut Hill, MA USA

**Keywords:** Perceived effects of exclusive breastfeeding, HIV-positive mothers, Maternal health, Infant health, Malawi

## Abstract

**Background:**

HIV-positive mothers are likely to exclusively breastfeed if they perceive exclusive breastfeeding (EBF) beneficial to them and their infants. Nevertheless, very little is known in Malawi about HIV-positive mothers’ perceptions regarding EBF. In order to effectively promote EBF among these mothers, it is important to first understand their perceptions on benefits of exclusive breastfeeding. This study therefore, explored maternal and health care workers’ perceptions of the effects of exclusive breastfeeding on HIV-positive mothers’ health and that of their infants.

**Methods:**

This was a qualitative study within a larger project. Face-to-face in-depth interviews and focus group discussions using a semi- structured interview and focus group guide were conducted. Sixteen HIV-positive breastfeeding mothers, between 18 and 35 years old, were interviewed and data saturation was achieved. Two focus group discussions (FGDs) comprising of five and six adult women of unknown HIV status who were personal assistants to maternity patients, and one FGD with five nurse-midwives working in the maternity wards of Queen Elizabeth Central Hospital in Blantyre, Malawi, were also conducted. Thematic content data analysis was utilized.

**Results:**

The study revealed more positive than negative perceived effects of exclusive breastfeeding. However, the fear of transmitting HIV to infants through breast milk featured strongly in the study participants’ reports including those of the nurse-midwives. Only one nurse-midwife and a few HIV-positive mothers believed that EBF prevents mother-to-child transmission of HIV. Furthermore, participants, especially the HIV-positive mothers felt that exclusive breastfeeding leads to maternal ill- health and would accelerate their progression to full blown AIDS.

**Conclusion:**

While most participants considered exclusive breastfeeding as an important component of the wellbeing of their infants’ health, they did not share the worldwide acknowledged benefits of exclusive breastfeeding in the prevention of mother-to-child transmission (PMTCT) of HIV. These results suggest a need for more breastfeeding education for all mothers, communities and nurse-midwives involved in breastfeeding counseling in the context of HIV infection. Maternal wellbeing promotion activities such as nutrition supplementation need to be included in all PMTCT of HIV programs.

**Electronic supplementary material:**

The online version of this article (doi:10.1186/1471-2393-14-247) contains supplementary material, which is available to authorized users.

## Background

Breastfeeding is an important component of the wellbeing and survival of children, particularly in resource-poor settings. Breast milk provides optimal nutrition, contains antibodies that protect infants from infection, and is unlikely to become contaminated [1–3]. These positive outcomes are more likely when exclusive breastfeeding is practiced [1].

Childhood diarrhea is among the top three causes of death in children globally [4]. In a review of data on child mortality, Black and colleagues [4] found that 41% of child deaths occur in Sub-Saharan Africa and 34% in South Asia. Additionally, the results revealed that 90% of all child deaths globally, occurred in 42 countries, which included Malawi. Unsafe environments, ingestion of unsafe water, inadequate availability of water for hygiene, and lack of sanitation constituted the major risk factors for childhood morbidity and mortality in these countries [4, 5]. In Malawi, the prevalence of diarrhea among children who are less than five years of age is 18%. For children who are less than two years of age the prevalence is almost double at 33% [5]. Most of such childhood diarrhea and subsequently deaths, could be prevented by exclusive breastfeeding [1, 3, 6].

Both local [7, 8] and international studies [1, 3, 6, 9, 10] have examined the relationship between exclusive breastfeeding and childhood and maternal health. Overall, exclusive breastfeeding was associated with significantly reduced childhood incidence of gastrointestinal [1, 10], respiratory [7], eye and malarial infections [7]. Furthermore, exclusively breastfed children had a reduced risk of mortality [3, 8, 9]. Exclusive breastfeeding provided a six-fold protection against gastrointestinal and almost a three-fold protection against acute respiratory infection in less developed countries [3]. In developing countries, exclusive breastfeeding for the first six months and then continued breastfeeding up to 11 months was associated with approximately 13% reduction in childhood deaths [6]. Similarly, in Latin America, Betran and colleagues [9] found that half of infant deaths from gastrointestinal and acute respiratory infections are preventable by exclusive breastfeeding.

The HIV and AIDS epidemic has complicated breastfeeding practice because breast milk carries HIV [11, 12]. In Malawi, mother-to-child transmission (MTCT) of HIV accounts for approximately 25% of new infections and breastfeeding is a significant mode of MTCT of HIV [13]. Although avoidance of breastfeeding can assist in preventing postnatal transmission of HIV in resource rich countries, this is not feasible for Malawi because the majority of women live below the poverty level [14]. As such, many mothers cannot afford infant formula. For those who might access infant formula, the risk of childhood morbidity and mortality from diarrhea and other non-HIV and HIV-related infections are real because of environmental circumstances that may not be safe and supportive of replacement feeding [4, 15]. Breastfeeding therefore remains the best way of feeding infants despite the risk of HIV infection and should be advocated for.

To the advantage of infants, the literature indicates that exclusive breastfeeding for the first six months of the infant’s life carries a 4-10 fold decreased risk of MTCT of HIV compared to mixed breastfeeding (feeding an infant other feeds besides its mother’s breast milk) [16, 17]. This may be due to the increased risk of diarrhea to the infant associated with unhygienic and unsafe environments and the use of unsafe water for formula preparation. Other foods and liquids alter the infant’s gastro-intestinal integrity and may therefore facilitate MTCT of HIV in mixed-fed infants [18].

Exclusive breastfeeding is also beneficial to the mother. In a Cochrane systematic review [1], exclusive breastfeeding was associated with a more rapid maternal postpartum weight loss and prolonged amenorrhea which may act as a family planning method. However, in qualitative studies from Sub-Saharan African countries [19–21], one of which studied HIV-positive mothers in Malawi [19], participants felt that EBF was demanding both physically on the mother’s body [19, 21] and on the mother’s time [20]. Mothers in a rural area of Cameroon [20] stated that they could not just sit down in order to exclusively breastfeed their infants without going to their fields to work. Likewise, mothers in Malawi [19] felt that exclusive breastfeeding would increase the progression of HIV. In Nigeria [21], mothers felt that their bodies were already weak from inadequate nourishment due to poverty, and that exclusive breastfeeding would weaken them further.

At the time of this study, postnatal recommendations for feeding HIV exposed infants in Malawi were exclusive breastfeeding for the first six months of infant’s life followed by abrupt weaning. Replacement feeding was supported only if it was acceptable, feasible, affordable, sustainable, and safe [22]. However, a more inclusive policy is currently in situ which expects all mothers, regardless of their HIV sero-status, to exclusively breastfeed for the first six months and then introduce complementary foods while continuing with breastfeeding until two years of the child’s life. For HIV-positive mothers, this policy is implemented alongside the lifelong anti-retro viral therapy (ART) option B + regime. With option B + regime, women who are HIV-positive are initiated on lifelong ART on the same day they are told about their results [23]. If properly implemented, option B + can reduce the risk of MTCT of HIV even through breastfeeding, to less than one percent [24]. In supporting the implementation of this policy, the Malawi Ministry of Health has embarked on nationwide in-service training of health care workers on integrated ART and PMTCT of HIV guidelines.

The way an individual perceives EBF may influence that individual’s practice of EBF [25]. A study from Ghana [26] indicated that mothers who had positive attitudes towards exclusive breastfeeding were twice as likely to exclusively breastfeed their infants than their counterparts (OR 2.0, 95% CI 1.11-3.57). A diverse range of views of mothers regarding the benefits of EBF on maternal and child’s health are well documented. Nevertheless, literature in Malawi is sparse on perceived benefits of EBF. This study therefore, was conducted to explore maternal and health care workers’ perceptions of the effects of exclusive breastfeeding by HIV positive mothers on maternal and infant health in Blantyre, Malawi.

## Methods

### Design

This was a qualitative study within a larger study that used in-depth interviews and focus group discussions to investigate mothers and health care workers’ perceptions of effects of exclusive breastfeeding on maternal and infant health in Blantyre, Malawi. The larger study utilized mixed methods and aimed at exploring culture-specific influences of exclusive breastfeeding among HIV-positive mothers in Blantyre, Malawi. In the current study, one-on-one in-depth interviews allowed HIV-positive mothers to participate while maintaining their privacy about their HIV status from other participants. Furthermore, in-depth interviews helped the researcher to explore the participants’ perceptions regarding effects of EBF on maternal and infant’s health. The focus group discussions helped the researchers to obtain comprehensive and diverse data in a short period of time. In addition, focus group discussions provided the researchers an opportunity to collect unanticipated data that would otherwise not have been collected through in-depth interviews. This study has adhered to the qualitative research review guidelines stipulated by BioMed Central.

### Study setting

This study was conducted at Queen Elizabeth Central Hospital (QECH) maternity unit in Blantyre, Malawi. Blantyre is situated in the southern region of Malawi. QECH is the largest public hospital in Malawi and functions both as a district hospital for Blantyre and as a referral hospital for the southern region of Malawi. It is also a teaching hospital for different health related professions. Most of the clients that come to QECH are of low and medium socioeconomic status because it is a public and heavily subsidized hospital. The maternity unit, normally known as Chatinkha maternity unit, is one of the busiest maternity units in Malawi with a bed capacity of 250. The unit conducts about 14000 deliveries per year of which 15% are delivered through cesarean section. The unit provides antenatal care to approximately 1,300 new mothers per year and a total of approximately 5,000 visits per year. Mothers who deliver in the unit and those who are referred from other hospitals are cared for in two postnatal wards. One of these postnatal wards is a paying ward for those mothers who can manage to pay and have decided to do so. The unit also has a well established PMTCT program that provides extra services to mothers who are HIV-positive and their infants. Furthermore, the unit provides family planning services within what is called postnatal clinic to approximately 2000 new clients per year [27]. Within QECH, participants were recruited from the postnatal wards, the postnatal clinic and the Cotrim clinic (a clinic that provided early infant HIV screening and cotrimoxazole to HIV exposed infants at the time of this study). Availability of well established PMTCT services provided access for the study of HIV-positive postnatal mothers without the researchers testing the participants for HIV.

### Sample and sampling

A purposive sample of 16 HIV-positive breastfeeding mothers, five (5) nurse-midwives and 11 adult women of unknown HIV sero-status was utilized. Purposive sampling allowed the researcher to recruit participants who were able to articulate their experience and provide adequate information on the research phenomenon. The actual sample size of 16 for the HIV-positive breastfeeding mothers was determined based on data saturation.

### Participant recruitment

One research assistant (RA) who was a nurse-midwife recruited participants. This RA was from a hospital other than the study setting to prevent coercion of participants. HIV-positive mothers who were at least18 years old, able and willing to give informed consent, able to understand and speak Chichewa and were breastfeeding a single child that was less than 6 months old were recruited. HIV-positive breastfeeding mothers who were sick with AIDS, tuberculosis or other medical conditions were excluded from the study. To qualify for the “women of unknown HIV status” category, one had to be at least 18 years old, of unknown HIV status, a personal assistant/guardian to a maternity patient at Chatinkha maternity unit at the time of the study, a mother with experience of breastfeeding a baby and not related to the participating HIV-positive mothers. These women were included to represent possible views of HIV-negative mothers, and potential mothers and mothers-in-law of HIV-positive mothers on exclusive breastfeeding. Some of the recruited HIV-positive mothers had not disclosed their HIV status to their mothers, mothers-in-law and spouses for fear of being stigmatized. Therefore, significant others of the recruited HIV-positive mothers were not included even though this would have been preferred. Nurse-midwives who had a minimum of two years of experience and who were involved in the care of HIV-positive prenatal and postnatal mothers at QECH during the time of data collection were eligible for the “nurse-midwives” category.

The RA introduced the study to potential participants. Recruitment scripts were utilized in order to maintain uniformity. HIV-positive mothers were recruited through the PMTCT program. No HIV testing and screening was conducted because all the mothers who were enrolled in the PMTCT program were HIV-positive. The women of unknown HIV status were recruited from QECH maternity unit guardian lounge while the nurse-midwives were recruited from QECH maternity wards.

### Data collection

Data were collected between April 16, 2009 and May 8, 2009. The researcher conducted all the audio-recorded IDIs and FGDs that lasted approximately one hour utilizing semi-structured guides. These IDIs and FGDs were conducted in a quiet private room to promote confidentiality of participants and quality of the audio-recorded data. One RA assisted the researcher with audio-tape recording of FGD sessions. The interview guide for HIV-positive mothers had prompts that elicited information regarding their age, parity, highest level of education reached and age of the infant being breastfed. In addition, the interview guide had a question on disclosure of HIV status. In order to assess participants’ perceived effects of EBF on maternal and infant’s health, all the participants were asked two questions: (1) What are some good things that would happen if you/an HIV-positive mother exclusively breastfed your/her baby? and (2) What are some bad things that would happen if you/an HIV-positive mother exclusively breastfed your/her baby? Participants were probed to elicit more information. Data collection for HIV-positive mothers continued until subsequent interview failed to elicit new information.

The two questions used to collect data were part of the semi-structured interview and focus group guides for a larger study that the current researchers conducted to explore culture specific influences of exclusive breastfeeding among HIV-positive mothers in Blantyre, Malawi. These guides were based on Theory of Planned Behavior [25]. The guides were validated by an expert in the area of breastfeeding in Malawi to ensure that they were culturally relevant and understandable to the study sample. Further refining of the guides was done once data collection had started in order to accommodate important emerging issues that the original prompts could not elicit. The main modification to the guides was on the local term used for exclusive breastfeeding because the participants came from different tribes and therefore gave different definitions of exclusive breastfeeding. This made the researchers to decide to give all the participants an operational definition of exclusive breastfeeding. This facilitated uniformity in the understanding of the participants on what exclusive breastfeeding is. See details of the guides in Additional file 1: In-depth Interview Guide; and Additional file 2: Focus Group Discussion Guide.

### Data management and analysis

Two transcribers knowledgeable in breastfeeding transcribed *verbatim* all IDI and FGD audio-tapes. Word by word transcription is helpful for quoting excerpts during data analysis and discussion [28]. The transcription was done in Chichewa (the language the IDIs and FGDs were conducted in). The researcher reviewed each transcription by comparing it to its original recorded version for accuracy. This process required listening to the audio-tapes and reading the transcriptions several times. The Chichewa transcriptions were then translated into English by one bilingual (Chichewa-English) person prior to data analysis.

Data analysis was done manually and concurrently with data collection in order to identify and correct errors during the subsequent interviews and focus group discussion, and to improve the guides by incorporating any emerging issues. Thematic content analysis was utilized [29] which was appropriate for this exploratory study. At the beginning of the analysis, the researcher read through one transcript quickly to get a sense of what was in it, and then read it again, this time closely and critically to identify codes that captured meaning [30]. The researcher discussed the initial coding scheme with experts in qualitative data analysis for validation of the accuracy of the codes. After incorporating any comments from the experts, the final coding scheme was then used to code the remaining transcripts from the IDIs and the FGDs. The codes were organized into mutually exclusive categories based on their similarities [29]. The different categories were then brought together to develop overarching themes represented by the different categories [31] which were presented as findings. A detailed record of all what was done was kept to serve as audit trail.

### Trustworthiness of data

Credibility was assured through prolonged immersion of the researcher into the situation, triangulation both by methods and source of data. Additionally, participants were encouraged through probes to share their perspectives without aiming for consensus. Dependability was assured by: (1) using the same semi-structured interview guide with each IDI and FGD although they were allowed to be flexible; (2) providing similar conditions with each group of participants; (3) preparing transcripts promptly; and (4) using direct quotes (the English translations of Chichewa quotes) when presenting findings. Confirmability was assured by providing a detailed description of what was done and decisions made which later served as an audit trail. Finally, transferability was assured by describing the sample and setting in detail so that potential appliers can make transferability decisions [32, 33].

### Ethical consideration

This study was part of the principle author’s academic work at New York University. It was important to get approval from the academic institution’s ethical body and from the site of the study. Therefore, the researchers sought approval from New York University Committee on Activities Involving Human Subjects (UCAIHS) and Malawi College of Medicine Research and Ethics Committee (COMREC) which was granted. Permission to access participants was obtained from the Director of QECH, the Head of Obstetric and Gynecology and Pediatric Departments of QECH. To avoid coercion of participants, health care providers of HIV-positive mothers did not directly participate in recruitment of participants. To help reduce stigmatization of FGD members, no names were used when sharing information and personal experiences were shared as if they were of someone else. Numbers were assigned to participants to be used when quoting information during data analysis. Each participant gave a written informed consent prior to participation.

## Results

### Description of sample

Sixteen HIV-positive mothers ranging in age from 18 to 35 years (mean of 27.7, s.d 5.326) were interviewed. Most of them had an academic level of less than Malawi School Certificate of Education (an academic qualification offered at the end of 4 years of successful high school education) and were not working (11/16 and 12/16 respectively). Half of them had disclosed their HIV status to their spouse or mother. The parity of the participants ranged from 1 to 6 with a mode of 1. More than half (9/16) of them were breastfeeding infants that were less than one week old.

Three focus group discussions were also conducted to complement data from the in-depth interviews. One focus group (FG) comprised of five female nurse-midwives who had been working in Chatinkha maternity wards for two and half to four years participated in the study. One of the nurse-midwives was in the age range of 25-29 years, three in 30-35 years and one in 45-55 years age range. There were no male nurse-midwives in Chatinkha Maternity Ward at the time of this study. Eleven adult women of unknown HIV sero-status who were assistants (guardians) of clients in the Chatinkha Maternity Unit of QECH participated in the two adult women focus group discussions of five and six participants each. Two of these women reported being in age range of 30-35 years and nine were in age range of 45-55 years. The two women in the age range of 30-35 year were still breastfeeding their children at the time of the study.

### Perceived effects of exclusive breastfeeding (EBF)

Statements on both positive and negative things that can happen if an HIV-positive mother exclusively breastfed her child emerged across all the study groups. Two categories of themes; *‘effects of exclusive breastfeeding on maternal health*’ and *‘effects of exclusive breastfeeding on child’s health’* emerged from these statements. Table 1 presents a summary of how the themes were derived from the data under each category.

**Table 1:** **Perceived effects of exclusive breastfeeding on maternal and infant health**

Categories	Themes	Quotes
Effects of exclusive breastfeeding on maternal health	• EBF promotes good nutritional habits for mothers.	• “Yes, there can be a good thing to the mother too if she exclusively breastfeeds in that she forces herself to eat more to produce more milk too”. (Interviewee_2_)
	• EBF is self-satisfying for the mother.	• “….the mother feels good if she exclusively breastfeeds her baby. You feel like you have done the best for your baby and you are happy”. (Interviewee_16_)
	• EBF prevents pregnancy.	• “… Some say that if one exclusively breastfeeds her baby, she does not get pregnant soon”. (FG1P_5_)
	EBF leads to maternal ill-health.	• “The mother’s body becomes weak if she exclusively breastfeeds her baby. She falls sick often too”. (FG2)
Effects of exclusive breastfeeding on infant health	• EBF promotes the well-being of the baby.	• “…A baby who is exclusively breastfed becomes strong, does not get sick often and if he gets diarrhea it does not take long before it is resolved…” (Intervieew_16_)
	• EBF is an expression of maternal love for her baby.	• “The baby will know that the mother loves him very much if she exclusively breastfeed him. It is a way of showing love to your baby”. (Interviewee_6_)
	• EBF prevents MTCT of HIV.	• “Exclusive breastfeeding can also help the baby not to get the virus because there will be no diarrhea if the baby continues to exclusively breastfeed because the virus cannot penetrate the gastro-intestinal tract”. (N-M_2_)
	• Breastfeeding transmits HIV to the baby.	• “…If the baby does not develop diarrhea while being breastfed, it will not contract the disease [HIV]… (Intervieew_13_)
		• “…it is not right for the HIV-positive mother to exclusively breastfeed her baby because the baby can get the HIV virus from the mother”. (Interviewee_15_)

### Effects of EBF on maternal health

Four subthemes of effects of EBF on maternal health emerged.

#### EBF promotes good nutritional habits for mothers

This subtheme emerged from HIV-positive mothers who felt that EBF provides an opportunity to HIV-positive mothers to boost their nutritional status. According to them, exclusive breastfeeding motivates mothers to eat more which in turn could help her to produce more milk for her baby. Eating more was viewed as a positive thing in these participants.

“Yes, there can be a good thing to the mother too if she exclusively breastfeeds in that she forces herself to eat more to produce more milk too. And also at the time of stopping breastfeeding [weaning the baby] and thereafter, you too can be encouraging yourself to eat a variety of foods when you are also giving these foods to the baby during the weaning process…this is good”. (Interviewee_2_)

#### EBF is self-satisfying for the mother

The belief that ‘EBF is self-satisfying for the mother’ also emerged from HIV-positive mothers only*.* These mothers reported that EBF is good because it gives HIV-positive mothers a sense of satisfaction regarding their motherhood experience.

“The other good thing is that the mother feels good if she exclusively breastfeeds her baby. You feel like you have done the best for your baby and you are happy”. (Interviewee_16_)

#### EBF prevents pregnancy

The belief that EBF prevents pregnancy emerged only from the adult women FG discussion. One participant reported that she has heard people say that EBF prevents pregnancy.

“Some say that if one exclusively breastfeeds her baby, she does not get pregnant soon. She will have time to breastfeed her baby before another pregnancy even if she slept with her husband”. (FG1P_5_)

#### EBF leads to maternal ill-health

This was the most common subtheme regarding the perceived effects of EBF on maternal health and was the third most common subtheme that emerged from the participating HIV-positive mothers. Participants felt that EBF is very demanding on the mother’s body. Participants also thought that EBF has the potential of weakening their health and making them develop AIDS faster than if they did not exclusively breastfeed. They also stated that EBF was bad because it can decrease the HIV-positive mother’s body immunity against diseases and the amount of blood in their bodies.

“The mother’s natural body protection can decrease if she decides to exclusively breastfeed her baby”. (Interviewee_4_)

“The problem is that you will breastfeed the baby exclusively for a long time but you will always have problems. Your body will not have enough blood”. (Interviewee_12_)

Furthermore, participants stated that EBF could lead to breast problems and frequent maternal illnesses.

“When one is exclusively breastfeeding a baby, it means the breast too is being compressed constantly. This can cause a problem. It is like you, compressing part of your body against a thorn. The thorn will prick you. In the same way the breast can develop blisters if it is constantly compressed in the baby’s mouth during breastfeeding”. (Interviewee_7_)

Similarly, participants from the adult women FG linked EBF to maternal ill-health. According to them, EBF weakens the mothers’ bodies and this makes them to fall sick frequently.

“The mother’s body becomes weak if she exclusively breastfeeds her baby. She falls sick often too”. (FG2)

### Effects of EBF on child health

Three subthemes of positive effects and one of negative effects of EBF on an infant’s health emerged.

#### EBF promotes the well-being of the baby

All the three groups of participants reported that EBF promotes the wellbeing of infants. This was the most common subtheme that emerged from the HIV-positive mothers regarding the effects of EBF on an infant’s health. Participants felt that EBF was an important component of an infant’s wellbeing. According to them, EBF encourages optimal growth and health of infants by protecting them from childhood diarrhea and recurrent episodes of other illnesses. Additionally, the participants believed that if infants who are exclusively breastfed suffer from diarrhea, they would recover from the diarrhea faster than those who are not exclusively breastfed.

“The good thing that can be there for my baby is that my baby will grow well and healthy and will not become malnourished. A baby who is exclusively breastfed becomes strong, does not get sick often and if he gets diarrhea it does not take long before it is resolved. This is so because the baby is receiving milk frequently and is strong at the time he is taken to the hospital for treatment”. (Interviewee_16_)

“If the mother does not exclusively breastfeed the baby, the baby will not benefit from the antibodies’ protection found in breast milk”. (N-M_2_)

“There is a good thing because the baby grows well and healthy. This is why I am saying this mother would want to exclusively breastfeed her baby so that her baby can grow well and healthy”. (FG2)

#### EBF is an expression of maternal love for her baby

Participants, except those from the nurse-midwives’ focus group, reported that mothers who choose to exclusively breastfed love their babies because it is a way of expressing maternal love to her baby.

“The baby will know that the mother loves him very much if she exclusively breastfeed him. It is a way of showing love to your baby”. (Interviewee_6_)

“Aaaa, I don’t see any bad thing because breastfeeding shows that the mother loves her baby and wishes the baby good growth”. (FG1P_1_)

“The baby gets ‘Big love’ from its mother”. (FG1P_3_)

#### EBF prevents MTCT of HIV

The belief that ‘*EBF prevents MTCT of HIV*’ emerged from few HIV-positive mothers and nurse-midwife focus group discussion. According to the HIV-positive mothers, choosing to exclusively breastfeed for six months gave them some hope of protecting their infants from contracting HIV from them. No participating adult woman felt that EBF prevents MTCT of HIV.

“If the baby is exclusively breastfed, it is difficult for the baby to get HIV virus from the mother because the baby breastfeeds only for six months. .. if longer than six months, the baby can get the virus”. (Interviewee_7_)

“Exclusive breastfeeding can also help the baby not to get the virus because there will be no diarrhea if the baby continues to exclusively breastfeed because the virus cannot penetrate the gastro-intestinal tract”. (N-M_2_)

In response to a follow up question on whether she was afraid of passing on the HIV infection to her infant through breastfeeding, one HIV-positive mother said:

“No, I am not afraid because they say one can breastfeed the baby for six months without giving the baby anything to eat so that the baby does not develop diarrhea. If the baby does not develop diarrhea while being breastfed, it will not contract the disease [HIV]. After six months, the baby should stop breastfeeding and the parents should buy milk”. (Interviewee_13_)

The protection that EBF provides against HIV was connected to not breastfeeding beyond six months of the infant’s age. Different explanations were given for why HIV-positive mothers should not breastfeed for more than six months.

“…some babies start developing teeth at this age therefore they can bite on the breast releasing some blood. If the blood drops into the baby’s mouth, and if the baby has some cuts in the mouth, then if the blood from the breast and that from the cuts touch each other, the baby can get the disease”. (Interviewee_1_)

“…After six months, the mother should wean the baby because at this time the baby has started receiving other foods and can therefore get HIV virus from its mother very easily”. (Interviewee_14_)

#### Breastfeeding transmits HIV to the baby

Almost all (15/16) HIV-positive mothers expressed fear of transmitting HIV to their infants, fear of having infants who are frequently sick and fear of death of their infants if they exclusively breastfed them. This was the second most common theme that emerged from HIV-positive mothers.

“The problem is also the same as for mothers that the baby will be getting sick often if exclusively breastfed because she will get the disease”. (Interviewee_12_)

To some of these mothers, the fear of transmitting the HIV infection to the infant undermined the benefits of exclusive breastfeeding to the infant. One HIV-positive mother who regarded EBF as important for her infant’s wellbeing also reported that:

“…it is not right for the HIV-positive mother to exclusively breastfeed her baby because the baby can get the HIV virus from the mother”. (Interviewee_15_)

Another HIV-positive mother was very explicit in her comment on the danger of HIV transmission as illustrated by the following statement:

“If we have the disease then that is it [tone of voice high]. We are going to transmit it to the baby”. (Interviewee_13_)

The magnitude of the participants’ fear of transmitting the HIV to the infant can be summarized by the following statement:

“Eeee [she paused and then looked at the researcher] it is dangerous because it is a risk since you don’t know if the baby will get it or not. Sometimes you don’t know how the baby’s blood test [HIV test] will turn out to be”. (Interviewee_6_)

The fear of transmitting HIV infection to an infant through breast milk also emerged from the nurse-midwives’ focus group discussion.

“The mother can pass the virus to her baby if she breastfeeds whether exclusively or not”. (N-M_3_)

Participants from the adult women FG also felt that breastfeeding could transmit HIV to infants and lead to infants’ ill-health.

“I think it is good for such a mother not to breastfeed the baby so that the mother does not transmit the disease to her baby”. (FG1)

“It is therefore good not to breastfeed the baby for fear of transmitting the disease [HIV infection] to the baby. Such a baby does not stay alive long”. (FG2)

## Discussion

The current study has revealed several effects of EBF by HIV positive mothers on maternal and infant health as perceived by mothers and midwives. Both positive and negative perceived effects emerged. Despite the HIV epidemic, mothers in the current study still regarded exclusive breastfeeding as an important component for an infant’s biophysical and psychological wellbeing. This finding was consistent with a previous study [34] in which mothers also felt that EBF promoted the child’s health. The mothers in our study were likely to exclusively breastfeed their infants because they perceived EBF as beneficial to their children [26]. However, the apparent fear of infecting their children with HIV through breast milk may be a real threat to the promotion of EBF among these mothers in Blantyre, Malawi. This finding may be related to the high knowledge level among women on MTCT of HIV. According to the Malawi Demographic Survey of 2010 [5], 91% of women and 86% of men know that breastfeeding can transmit HIV. Furthermore, the same demographic health survey shows that 86% of women and 78% of men in Malawi know that the risk of MTCT of HIV can be reduced if the mother takes ART during pregnancy. The latter finding is encouraging especially considering the advent of the current ART option B + regime which greatly reduces the risk of MTCT of HIV. With comprehensive and individualized counseling on infant feeding that includes the role of option B + regime in prevention of MTCT of HIV, the recommended infant feeding practices in Malawi could be accomplished because more HIV-positive mothers would choose to breastfeed their children without much fear.

The fear of MTCT of HIV through breastfeeding was not unique to the Blantyre participants. Earlier studies from the sub-Saharan region have also revealed that both health care workers [35] and mothers [36–38] felt that breastfeeding is a risky way of feeding children in the presence of HIV infection because children can contract the virus from the mother.

In our study, the nurse-midwives’ statements regarding the feeding of infants of HIV-positive mothers reflect views about the ideal situation in which the surest way of preventing MTCT of HIV is to abstain from breastfeeding. However, this idealistic approach is not practical for a resource-poor country like Malawi where majority of HIV-positive mothers cannot afford replacement feeding. In such a setting, a balance of benefits and disadvantages of breastfeeding is a better guiding principle in choosing the type of infant feeding option. Concurring, Leshabari and colleagues’ study of nurse counselors in Northern Tanzania on HIV and infant feeding options found that nurse counselors were ambivalent about the appropriateness of the feeding options for HIV-positive mothers because they regarded both EBF and replacement feeding (RF) as culturally and socially unsuitable. In this culture, mixed feeding is the acceptable mode of infant feeding [35]. However, as in the current study, the nurse counselors in Leshabari and colleagues’ study believed that formula feeding was the right option for HIV-positive mothers because it eliminates the risk of MTCT of HIV through breast milk. For a country like Malawi, where the majority of HIV-positive mothers are poor, failure to exclusively breastfeed infants could risk these infants to high morbidity and mortality associated with replacement feeding and malnutrition due to over dilution of formula in cases of inadequate formula and infection due to unhygienic environment. Our findings therefore, support the need to educate health care workers, HIV-positive mothers and society on the role of EBF in the promotion of an infant’s wellbeing. With the advent of option B + (a life-long HIV triple therapy initiated at the earliest possible time of diagnosis prenatally or postnatally) in Malawi, that is aimed at slowing progression, increasing survival and reducing MTCT of HIV [23], health care workers need to be helped to change their mindset regarding infant feeding options for them to be able to appropriately counsel mothers who are expected to breastfeed until the child is 24 months old. The current ongoing in-service trainings on integrated ART and PMTCT of HIV should be mandatory for all health care workers involved in the care of pregnant and breastfeeding mothers in Malawi.

Although not supported by the nurse-midwives, the HIV-positive mothers and the adult women of unknown HIV status considered EBF important in building an intimate relationship between a mother and her infant. The fact that EBF requires the mother-infant dyad to be together most of its time may indeed facilitate bonding between the two through the frequent intimate interactions that take place during each feeding [39, 40].

Other highlighted perceived benefits of EBF include a sense of satisfaction, delay in subsequent pregnancies and promotion of optimal maternal dietary intake which in these participants was viewed as a positive thing. Consistent with these findings were those from previous studies [34, 40] in which participants linked EBF with lactational amenorrhea, and improved maternal appetite. Approximately 79% of the participants in Uchendu and colleagues’ study [34] reported that mothers who exclusively breastfeed their infants eat more. This finding may be helpful in the promotion of maternal health and EBF among HIV-positive mothers. Mothers with a good appetite are more likely to have adequate food intake as long as food is available to them.

However, of great concern to the promotion of EBF among HIV-positive mothers is the participants’ belief that ‘EBF leads to maternal ill-health’. This belief may deter HIV-positive mothers from exclusively breastfeeding their infants for fear of accelerating the development of their HIV infection to full blown AIDS. This finding concurs with that from an earlier study of HIV-positive mothers in Lilongwe, Malawi, in which mothers believed that EBF would enhance the progression of HIV to AIDS [19]. Similarly, HIV-positive mothers in a South Africa study [38] also felt that EBF was a burden on them. These mothers’ concerns about the disadvantages of EBF on their health could be genuine considering the findings from a study that was conducted in Nigeria by Okechuku and colleagues [41]. Okechuku and colleagues studied breastfeeding mothers from a general population to evaluate the effects of EBF on their anthropometry during the first six weeks after birth. Although their findings revealed no major nutritional status differences (based on the weight/height Z scores, an index of thinness and body mass index that determines the nutritional status of an individual) between mothers who exclusively breastfed their infants and those who did not, the results revealed a significantly high body weight loss, significant loss in mid-arm circumference (MAC) and significant net loss of triceps skin-fold thickness (TST) among mothers who exclusively breastfed compared to those who did not EBF their babies (P < 0.05). Kramer and Kakuma [1] also revealed that rapid loss of postpartum weight occurred in mothers who exclusively breastfed their infants. However, studies of HIV-positive breastfeeding mothers from the sub-Saharan region including Malawi, revealed that maternal mortality and morbidity are neither significantly correlated to breastfeeding [8, 42–44] nor to the exclusivity of the breastfeeding [8, 44]. Despite the strong evidence from previous studies regarding the absence of disadvantages for HIV mothers who exclusively breastfeed, it is important to note that most of the HIV-positive mothers that choose to EBF their babies in Malawi are poor [14] and may have problems in finding adequate and appropriate food for themselves. In view of this fact and as suggested by Bentley and colleagues [19], promotion of appropriate infant feeding options should also focus on maternal nutrition and wellbeing because the nutritional demands that EBF impose on HIV-positive mothers as revealed by previous studies [1, 41] could be adverse in HIV-positive mothers who are already malnourished. Furthermore, the psychological stress associated with constant fear and worry about their health including that of how to find food may affect these mothers’ milk letdown reflex, breast emptying and subsequent milk production [45]. This in turn may reduce the success rate of exclusive breastfeeding in these mothers because of the resultant breast milk insufficiency.

## Conclusion

The findings of this study have revealed both positive and negative perceived effects of exclusive breastfeeding on maternal and infant health. Although there were more themes of positive than negative perceived effects of exclusive breastfeeding on maternal and infant health, the two negative themes (EBF promotes maternal-ill health’ and ‘EBF promotes mother-to-child transmission of HIV’) that emerged may counteract efforts in the promotion of exclusive breastfeeding among HIV-positive mothers. However, the introduction of the lifelong ART (option B+) may help to reduce these fears as it greatly decreases the risk of MTCT of HIV and slows the progression of HIV to full blown AIDS. The applicability of the findings from the present study may be limited because participants were drawn from one site that caters mainly for low income women. It is not known what HIV-positive mothers of high socio-economic status would perceive as effects of EBF on their health and that of their infants. Further research with a more diverse sample is therefore recommended to explore maternal and health care workers’ perceptions regarding benefits of exclusive breastfeeding on mothers and their infants. Despite this limitation, these findings suggest a need for more breastfeeding education that takes into consideration current policy guidelines for feeding HIV exposed infants. Pre-service curriculum for all health care workers should include ART and PMTCT components so that all providers are appropriately equipped with knowledge and skills to counsel mothers on breastfeeding in line with the recommendations in situ. Provide comprehensive information and education on lifelong ART (option B+) regime to al health care workers and mothers to alleviate fears associated with MTCT of HIV through breast milk. Clear linkages between PMTCT programs and pre-existing nutrition rehabilitation centers within Blantyre, Malawi should be put in place. This would facilitate timely and appropriate referrals of any underweight or malnourished HIV-positive mothers in need of nutritional supplementation.

## Electronic supplementary material

Additional file 1: **In-depth Interview Guide.** (DOC 42 KB)

Additional file 2: **Focus Group Discussion Guide.** (DOC 40 KB)

## Abbreviations

EBF: Exclusive breastfeeding
FGD: Focus group discussion
FGDP: Focus group discussion participant
FG1P_3_: Focus group one participant three
IDI: In-depth interview
MAC: Mid-arm circumference
MTCT: Mother-to-child transmission
PMTCT: Prevention of mother-to-child transmission
QECH: Queen Elizabeth Central Hospital
RA: Research assistant.
