# Supplementary material for: Maternal and health care workers’ perceptions of the effects of exclusive breastfeeding by HIV positive mothers on maternal and infant health in Blantyre, Malawi
Source: BMC Pregnancy Childbirth. 2014 Jul 25;14:247. doi: 10.1186/1471-2393-14-247 (PMC4119201; doi:10.1186/1471-2393-14-247)
Supplement: Supplementary file 1 — Additional file 1: In-depth Interview Guide. (DOC 42 KB) [file 12884_2013_1115_MOESM1_ESM.doc]

In-depth Interview Guides- English versions

**Main Study Title:** Culture-specific influences of Exclusive Breastfeeding among HIV-positive mothers in Blantyre, Malawi

**Sub-study Title:** Maternal and Health Care workers' perceptions of the effects of exclusive breastfeeding by HIV positive mothers on maternal and infant health in Blantyre, Malawi

**NB:** Data for this sub-study is based on interview guide questions 4 and 5.

#### An In-depth Interview Script for HIV-positive Mothers

**Materials Needed:**

- 2 recorders (for back-up)
- 2 copies of consent form for each participant
- A copy of interview guide for HIV-positive mothers and a print out of 1 guide question per page to facilitate note taking
- An ink pad for thumb print signing of consent form.
- Pens for writing notes

*A. Introductions and Interview Process (5 minutes)*

1. Welcome the participant as she arrives at the interview venue. Get her seated in a chair in a quiet and private place. The researcher should also sit in a similar chair (if on the floor, all should sit on the floor).

2. The researcher introduces herself.

3. Ask the participant to introduce herself by saying her first name only, why she is here today, and what she would be doing if she were not here.

4. Explain the purpose of the interview session by saying:

Welcome to today’s interview session. I am planning to gather information that would later help me to develop an HIV prevention program for mothers like you who intend to breastfeed their babies in Blantyre. I will also use the information that I will collect to write my academic paper (dissertation) in partial fulfillment of my degree at New York University, USA. I asked you to come to today’s session because I would like to hear from you about your opinions and ideas on exclusive breastfeeding for HIV-positive mothers. You are the expert, and I can learn from you. I need your honest opinion – good and bad – about exclusive breastfeeding when a mother is HIV-positive. I would like you to share what you think and what you think other mothers who are HIV-positive in Blantyre think about exclusive breastfeeding. I would like to take what you say and put together for my academic paper and also develop a program for HIV-positive mothers who can not afford replacement feeding for their babies to help them reduce the chances of infecting their babies with HIV virus. Everything you are thinking is important to me. There are no right or wrong answers. I value your opinion. I would be very happy if you would help me to make the best program possible.

5. Consent forms: Review the consent form with the participant, answer any questions, obtain signature/thumb prints on one copy of consent form, give unsigned copy to participant, and return the signed copy to the lockable cupboard kept at Kamuzu College of Nursing, P. O. Box 415, Blantyre, Malawi.

**B. In-depth Interview Guide (45 minutes):**

Participant Number:

Date of interview:

**Demographic Data**

Age of participant:

Highest level of education reached:

Parity:

Age of infant being breastfed:

To who have you disclosed your HIV status? ---------------------------------------

**Information on exclusive breastfeeding**

Elicit information on exclusive breastfeeding when a mother is HIV-positive by asking the following questions. The researcher will conduct the interview and manage the audio taping of the interview using 2 audio-tapes. Any notes should be written down on the separate print out of each guide question.

**NB:** The operational definition of exclusive breastfeeding for this study should be given to each participant after question #1 and before proceeding with the rest of the interview. In this study, exclusive breastfeeding is defined as feeding an infant only breast milk from his/her mother, and no other liquids or solids with the exception of drops or syrups consisting of vitamins, mineral supplements, or medicines. This will be done to facilitate the understanding of the subsequent questions and also to make sure that all participants will be responding to the same questions.

1. What does exclusive breastfeeding mean to you?

2. Do you think HIV-positive mothers should exclusively breastfeed their babies? Why or why not?

3. Do you think you would want to exclusively breastfeed your baby? Why or why not?

4. What are some good things that could happen if you or any HIV-positive mother exclusively breastfeed your/her baby?

**Probe** for good things for both the baby and the mother/family.

5. What are some bad things that could happen if you or any HIV-positive mother exclusively breastfeed your/her baby?

**Probe** for bad things for both the baby and the mother/family.

6. What would make it easier for you to exclusively breastfeed your baby?

**Probe** for resources like her commitment, knowledge and experience about exclusive breastfeeding, time to exclusively breastfeed her baby etc.

**Probe** for socio-cultural issues such as cultural practices associated with childcare and feeding.

**Probe** for systems factors such as commitment of health care providers, consistency in type of information provided to HIV-positive mothers about exclusive breastfeeding, and communication and continuity of support for HIV-positive mother after discharge from maternity unit.

7. What would make it harder for you to exclusively breastfeed your baby?

**Probe** for resources like her commitment, knowledge and experience about exclusive breastfeeding, time to exclusively breastfeed her baby, adequacy of breast milk, etc.

**Probe** for socio-cultural issues such as cultural practices associated with childcare and feeding, appropriateness of breast milk during different maternal health conditions.

**Probe** for systems factors such as commitment of health care providers, consistency in type of information provided to HIV-positive mothers about exclusive breastfeeding, and communication and continuity of support for HIV-positive mother after discharge from maternity unit.

8. Who would approve of you to exclusively breastfeed your baby?

**Probe** for decision makers on issues concerning childcare and feeding.

9. Who would disapprove of you to exclusively breastfeed your baby?

**Probe** for decision makers on issues concerning childcare and feeding.

10. Who else would influence you to exclusively breastfeed your baby?

11. Are there any other thoughts you have about exclusive breastfeeding while HIV-positive that I haven’t asked you?

1. **Interview Observations**- **To be done by the interviewer (take brief notes where possible without interrupting the flow of the interview**

The observations of the interview should include the following:

- 1. Forthcoming of the participant in responding to the questions and probes.
  2. Tone of the interaction (angry, despair, bored, etc).
  3. Consistency of participant’s responses.
  4. Body language.

**D. Wrap Up (5 minutes)**

I greatly appreciate all of your thoughts and ideas. You have been a big help, and I want to thank you very much for all the information you have shared with me today. I know that your ideas will help me to write my academic paper and develop effective interventions to help mothers who may not afford replacement feeding for their babies. Thank you once again for your participation in this study – I really appreciate you!

Adapted from the Jamaican Mother-Daughter HIV Risk Reduction Project (Hutchinson, et al., 2007)
