# Supplementary material for: Maternal and health care workers’ perceptions of the effects of exclusive breastfeeding by HIV positive mothers on maternal and infant health in Blantyre, Malawi
Source: BMC Pregnancy Childbirth. 2014 Jul 25;14:247. doi: 10.1186/1471-2393-14-247 (PMC4119201; doi:10.1186/1471-2393-14-247)
Supplement: Supplementary file 2 — Additional file 2: Focus Group Discussion Guide. (DOC 40 KB) [file 12884_2013_1115_MOESM2_ESM.doc]

Focus Group Discussion Guide

**Main Study Title:** Culture-specific influences of Exclusive Breastfeeding among HIV-positive mothers in Blantyre, Malawi

**Sub-study Title:** Maternal and Health Care workers' perceptions of the effects of exclusive breastfeeding by HIV positive mothers on maternal and infant health in Blantyre, Malawi

**NB:** Data for this sub-study is based on focus group discussion guiding questions 4 and 5.

#### Focus Group Script for Adult Women and Nurses and Midwives.

**Materials Needed:**

- 2 recorders (for back-up)
- 2 copies of consent form for each participant
- A copy of focus group guide and a print out of 1 guide question per page to facilitate note taking
- An ink pad for thumb print signing of consent form.
- Pens for taking notes

*A. Introductions and Focus Group Process (10 minutes)*

1. Welcome participants as they arrive at the focus group venue. Get them seated around a table. The researcher should also sit around the same table. The research assistant should sit outside the table so that her note taking does not disturb the group dynamics.

2. The researcher and research assistant introduce themselves.

3. Ask the participants to introduce themselves by saying their first name only, why they are here today, and what they would be doing if they were not here.

4. Explain the purpose of the focus group session by saying:

Welcome to today’s focus group discussion. I am planning to gather information that would later help me to develop an HIV prevention program for HIV-positive mothers who intend to breastfeed their babies in Blantyre. I will also use the information to write my academic paper (dissertation) in partial fulfillment of my degree at New York University, USA. I asked you to come to today’s session because I would like to hear from you about your opinions and ideas on exclusive breastfeeding in HIV-positive mothers. You are the experts, and I can learn from you. I need your honest opinion – good and bad – about exclusive breastfeeding when a mother is HIV-positive. I would like you to share what you think – and what you think other nurses, midwives, or (adult men and women) in Blantyre think about exclusive breastfeeding when a mother is HIV-positive. I would like to take what you say and put together for my dissertation and later develop a program for HIV-positive mothers who can not afford replacement feeding for their babies to help them reduce the chances of infecting their babies. Everything you are thinking is important to me. There are no right or wrong answers. I value your opinion. I would be very happy if you would help me to make the best program possible.

5. Consent forms: review the consent form with the participants, answer any questions, obtain signature/thumb print on one copy of consent form, give one unsigned copy to each participant, and return the signed copies to the lockable cupboard kept at Kamuzu College of Nursing, P.O Box 415, Blantyre, Malawi.

***B. Group Rules (5 minutes)***

1. Develop group rules to protect participants’ confidentiality. Examples of group rules:

- Be respectful
- No put downs or insults
- One person at a time speaks
- Be honest
- Maintain confidentiality

**“**Although I do not ask about YOUR individual exclusive breastfeeding experience, I do ask about your opinion about exclusive breastfeeding when a mother is HIV-positive in general. Because this is a sensitive issue, I do not want you to talk about your own specific experience or name anyone else who you are talking about.  That would violate your privacy and/or the other person’s privacy. If you want to talk about your experience with exclusive breastfeeding, PLEASE DO NOT SAY THAT THESE ARE THINGS THAT YOU YOURSELF EXPERIENCED AND PLEASE DO NOT NAME ANYONE YOU ARE TALKING ABOUT***.***

***C. Focus Group Discussion (45 minutes):***

Number of participants in the group:

**Demographic data**

Age of each participant and any special characteristic:

Participant 1:

Participant 2:

Participant 3:

Participant 4:

Participant 5:

etc

**Information on exclusive breastfeeding**

Elicit information on exclusive breastfeeding when a mother is HIV-positive by asking the following questions. The research assistant should facilitate the audio-taping of the discussions using 2 audio-tapes, and observe focus group dynamics. All of the observations should be written on the separate print out of each guide question.

**NB:** The operational definition of exclusive breastfeeding for this study should be given to each participant after question #1 and before proceeding with the rest of the interview. In this study, exclusive breastfeeding is defined as feeding an infant only breast milk from his/her mother, and no other liquids or solids with the exception of drops or syrups consisting of vitamins, mineral supplements, or medicines. This will be done to facilitate the understanding of the subsequent questions and also to make sure that all participants will be responding to the same questions.

1. What does exclusive breastfeeding mean to you?

2. Do you think HIV-positive mothers should exclusively breastfeed their babies? Why or why not?

3. Do you think HIV-positive mothers would want to exclusively breastfeed their babies? Why or why not?

4. What are some good things that could happen if HIV-positive mothers exclusively breastfeed their babies?

**Probe** for good things for both the baby and the mother/family.

5. What are some bad things that could happen if HIV-positive mothers exclusively breastfeed their babies?

**Probe** for bad things for both the baby and the mother/family.

6. What makes it easier for HIV-positive mothers to exclusively breastfeed their babies?

**Probe** for resources like commitment of HIV-positive mothers, knowledge and experience about exclusive breastfeeding, time to exclusively breastfeed the baby etc.

**Probe** for socio-cultural issues such as cultural practices associated with childcare and feeding.

**Probe** for systems factors such as commitment of health care providers, consistency in type of information provided to mothers about exclusive breastfeeding when a mother is HIV-positive, and communication and continuity of support when the mother is discharged from the maternity unit.

7. What makes it harder for HIV-positive mothers to exclusively breastfeed their babies?

**Probe** for resources like commitment of HIV-positive mothers, knowledge and experience about exclusive breastfeeding, time to exclusively breastfeed the baby, adequacy of breast milk, etc.

**Probe** for socio-cultural issues such as cultural practices associated with childcare and feeding, adequacy and appropriateness of breast milk during different maternal health conditions.

**Probe** for systems factors such as commitment of health care providers, consistency in type of information provided to mothers about exclusive breastfeeding when the mother is HIV-positive, and communication and continuity of support when the mother is discharged from the maternity unit.

8. Who would approve of HIV-positive mothers to exclusively breastfeed their babies?

**Probe** for decision makers on issues concerning childcare and feeding.

9. Who would disapprove of HIV-positive mothers to exclusively breastfeed their babies?

**Probe** for decision makers on issues concerning childcare and feeding.

10. Who else would influence HIV-positive mothers to exclusively breastfeed their babies?

11. Are there any other thoughts you have about exclusive breastfeeding when a mother is HIV-positive that I haven’t asked you?

1. ***Focus Group Observations - To be done by the research assistant***

The observations of the group dynamics should include the following:

- 1. Who participates in the discussion and who does not
  2. Who dominates in the discussion
  3. Tone of the group interaction (collegial, conflicted, angry, bored, etc)
  4. Consistency of participants’ observations.
  5. Body language

***E. Wrap Up (5 minutes)***

I greatly appreciate all of your thoughts and ideas. You have been a big help, and I want to thank you very much for all the information you have shared with me today. I know that your ideas will help to write my dissertation and develop effective interventions to help HIV-positive mothers who cannot afford replacement infant feeding for their babies. Thank you once again for your participation – I really appreciate you!

Adapted from the Jamaican Mother-Daughter HIV Risk Reduction Project (Hutchinson, et al., 2007)
